# Pontine Infarction Presenting With Right Internuclear Ophthalmoplegia in a Patient With Patent Foramen Ovale and May-Thurner Syndrome: A Case Report

**DOI:** 10.7759/cureus.115744

**Published:** 2026-09-03

**Authors:** Khoa Pham, Pho Doan, Long Nguyen, Paul Parvesh, Max Rash

**Affiliations:** 1 Internal Medicine, William Carey University College of Osteopathic Medicine, Hattiesburg, USA; 2 Internal Medicine, North Mississippi Medical Center, Tupelo, USA

**Keywords:** atrial septal aneurysm, cryptogenic strokes, internuclear ophthalmoplegia, patent foramen ovale (pfo), pontine infarct

## Abstract

Patent foramen ovale (PFO) is a common congenital cardiac anomaly associated with cryptogenic ischemic stroke in adults. The likelihood of PFO-associated stroke is increased in the presence of high-risk anatomical features such as substantial right-to-left shunting or an associated atrial septal aneurysm (ASA). Brainstem ischemic events associated with PFO, however, are less frequently reported. We present the case of a 59-year-old man who developed dizziness, binocular diplopia, right internuclear ophthalmoplegia (INO), and gait instability. Magnetic resonance imaging demonstrated an old pontine infarct without evidence of recent or acute infarction; however, based on the acute clinical presentation and neurologic examination, neurology considered the presentation clinically consistent with a subacute pontine ischemic infarct. Subsequent cardiac evaluation demonstrated a PFO with right-to-left shunting and an associated ASA, and the patient later underwent successful percutaneous PFO closure. The coexistence of May-Thurner syndrome (MTS) provided a potential venous thrombotic substrate for paradoxical embolism, although no active venous thrombus was documented. This case highlights an uncommon brainstem presentation in a patient with high-risk PFO anatomy and emphasizes the importance of distinguishing a plausible paradoxical embolic mechanism from proven causation.

## Introduction

Cryptogenic ischemic stroke is defined as a symptomatic cerebral infarction in which no probable etiology is identified after a standard diagnostic evaluation [1]. Cryptogenic strokes account for approximately 25-30% of ischemic strokes, and a patent foramen ovale (PFO) is identified in a substantial proportion of affected younger and middle-aged adults [2]. Although PFO is common in the general population, certain anatomical characteristics, including an associated atrial septal aneurysm (ASA) and substantial right-to-left shunting, increase the likelihood that the PFO may be clinically relevant in patients with otherwise unexplained ischemic stroke [2,3].

The foramen ovale is a normal fetal interatrial communication that allows oxygenated placental blood to bypass the pulmonary circulation. Functional closure generally occurs after birth when left atrial pressure exceeds right atrial pressure. Failure of complete anatomical fusion results in persistence of a PFO, which is present in approximately 25% of adults [2]. Although most PFOs are clinically silent, transient increases in right atrial pressure may permit right-to-left shunting and create a potential pathway for venous material to enter the systemic arterial circulation.

May-Thurner syndrome (MTS) is caused by compression of the left common iliac vein by the overlying right common iliac artery, resulting in impaired venous outflow and an increased risk of venous thrombosis [4,5]. In patients with a PFO and right-to-left shunting, this venous thrombotic predisposition may provide a potential substrate for paradoxical embolism. However, the coexistence of MTS and PFO alone does not establish a causal embolic mechanism.

Ischemic strokes potentially attributable to PFO more commonly demonstrate cortical involvement, whereas brainstem presentations in patients with PFO are less frequently described. Brainstem infarctions represent a minority of ischemic strokes; in the Lausanne Stroke Registry, 113 of 891 cerebral infarctions involved the brainstem [6]. Common etiologies include large-artery atherosclerosis, cardioembolism, small-vessel disease, and vertebrobasilar arterial pathology [7]. In a rehabilitation cohort of patients with brainstem stroke, pontine lesions accounted for approximately 40% of lesions [8].

Internuclear ophthalmoplegia (INO) is a characteristic ocular motor syndrome resulting from disruption of the medial longitudinal fasciculus [9,10]. Although ischemia is a well-recognized cause of INO in older adults, brainstem ischemic presentations predominantly manifesting as INO in patients with PFO are infrequently described. We present a patient with acute right INO and gait instability who was clinically diagnosed with a subacute pontine ischemic infarct despite MRI findings interpreted as chronic, and whose subsequent evaluation demonstrated a PFO with right-to-left shunting and ASA. This case highlights an uncommon neurologic presentation and the diagnostic challenge of determining the significance of a PFO in a clinically suspected brainstem ischemic event.

## Case presentation

A 59-year-old man presented to the emergency department with dizziness, binocular diplopia, and gait instability, with a tendency to fall toward the left. His symptoms began approximately nine hours before presentation. His past medical history was significant for splenic artery aneurysm, cutaneous angiokeratoma, testicular hypofunction, chronic venous insufficiency, mast cell disease, and MTS. Hypertension and diabetes were not documented in the available medical history, and he had no smoking history. The patient reported a similar episode five months earlier that required hospitalization; however, a formal diagnosis of stroke or transient ischemic attack from that episode was not documented in the available records. During both episodes, he reported that his visual symptoms subjectively improved with rightward gaze.

On physical examination, vital signs were within normal limits, including a blood pressure of 125/84 mmHg, heart rate of 84 beats per minute, respiratory rate of 16 breaths per minute, oxygen saturation of 94% on room air, and a temperature of 97.7°F.

Neurologic examination demonstrated an alert and fully oriented patient with fluent speech. Pupils were equal, round, and reactive to light. Extraocular movement examination demonstrated right INO with right exotropia and impaired adduction of the right eye during leftward gaze. No abducting nystagmus was observed. Elevation and depression of both eyes were preserved. Convergence was not formally assessed, and a specific assessment for skew deviation was not documented. The initial cranial nerve examination raised concern for minimal left facial weakness; however, no definite persistent facial weakness was documented on subsequent neurologic evaluation. The remainder of the cranial nerve examination was unremarkable. Motor strength, sensation, appendicular coordination, and deep tendon reflexes were within normal limits.

Initial laboratory evaluation was unrevealing. Troponin I was < 0.012 ng/mL. A complete thrombophilia or hypercoagulability panel was not documented in the available records.

Non-contrast computed tomography (CT) of the head showed no acute intracranial abnormality. Computed tomography angiography (CTA) of the head and neck demonstrated no large-vessel occlusion, arterial dissection, or hemodynamically significant stenosis.

**Figure 1:**
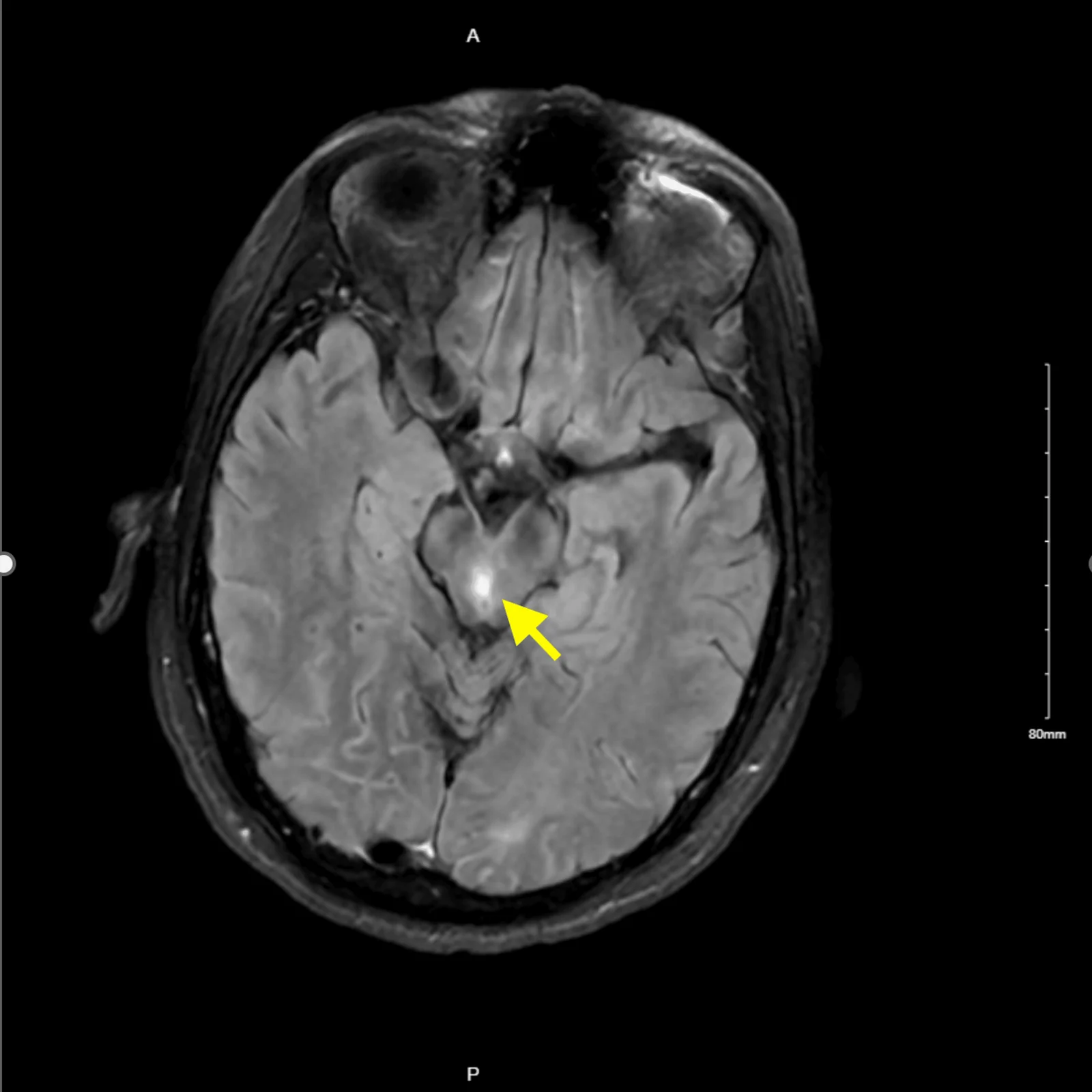
Axial fluid-attenuated inversion recovery (FLAIR) MRI demonstrating a hyperintense focus in the right paramedian pons (yellow arrow), corresponding anatomically to the region of the medial longitudinal fasciculus. The formal radiology interpretation described the pontine lesion as an old infarct and reported no evidence of recent or acute infarction. MRI: magnetic resonance imaging

Magnetic resonance imaging (MRI) of the brain showed no evidence of recent or acute infarction. Scattered subcortical and periventricular white matter abnormalities were interpreted as changes related to chronic small-vessel disease. On axial fluid-attenuated inversion recovery (FLAIR) imaging, a hyperintense focus was present in the right paramedian pons (Figure 1), corresponding to the lesion interpreted by radiology as an old pontine infarct. Despite the absence of a radiographically acute lesion, neurology considered the patient's acute clinical presentation and ocular motor findings consistent with a subacute pontine ischemic infarct manifesting predominantly as right INO and diplopia. Electrocardiography demonstrated normal sinus rhythm.

The patient was admitted for further neurologic evaluation and treatment of suspected ischemic stroke. Transthoracic echocardiography demonstrated a PFO with predominant right-to-left shunting and an associated ASA. The available echocardiographic documentation did not provide a quantitative bubble count or clearly specify whether shunting was present at rest or only with a Valsalva maneuver.

The patient was initiated on dual antiplatelet therapy with aspirin and clopidogrel. His neurologic symptoms remained stable without progression, and he was discharged with plans for additional outpatient evaluation of the PFO.

One week after discharge, transesophageal echocardiography confirmed a PFO with right-to-left shunting and an associated ASA. The available report did not provide a quantitative shunt grade or sufficient detail regarding rest versus Valsalva-provoked shunting.

Given the recurrent neurologic symptoms and high-risk PFO anatomy, the patient subsequently underwent percutaneous PFO closure. Intracardiac echocardiography and fluoroscopic guidance were used during the procedure, and a 30-mm GORE CARDIOFORM Septal Occluder (W. L. Gore & Associates, Inc., Flagstaff, Arizona, USA) was successfully deployed without immediate complications.

During the procedure, the patient’s previously placed left iliac vein stent for May-Thurner syndrome was noted to be incompletely apposed. No lower-extremity duplex ultrasound or pelvic CT/MR venographic study from the index hospitalization was documented in the records available to the authors.

## Discussion

Several mechanisms have been proposed to explain the association between PFO and ischemic stroke. The most widely recognized is paradoxical embolism, in which venous material traverses a right-to-left intracardiac shunt and enters the systemic arterial circulation [11]. Other proposed mechanisms include thrombus formation within the PFO tunnel or interatrial septal region and potential associations with atrial arrhythmias [11]. However, because PFO is common in the general population, its identification after an ischemic neurologic event does not by itself establish causality. In this patient, the coexistence of right-to-left shunting and an ASA makes a PFO-associated mechanism clinically plausible, but not definitive.

The patient's MTS represents a potential venous thrombotic substrate, or “thrombotic engine,” for paradoxical embolism. MTS may promote iliac venous stasis and increase the risk of thrombosis and has been reported in patients with cryptogenic stroke and PFO [4,5,12]. Although no active venous thrombus was documented in this patient, the coexistence of MTS, PFO with right-to-left shunting, and ASA provides a biologically plausible pathway for paradoxical embolization.

The clinical-radiologic discrepancy in this case is important. The formal MRI interpretation demonstrated an old pontine infarct with no evidence of recent or acute infarction, while the treating neurologist considered the current clinical syndrome consistent with a subacute pontine ischemic infarct based on the patient's symptoms and ocular motor findings. The patient also reported a similar episode five months earlier, raising the possibility that the chronic pontine lesion was related to the previous event. Therefore, the imaging abnormality cannot be definitively attributed to the current presentation. In addition, convergence was not formally assessed, and skew deviation was not specifically documented during the ocular motor examination, limiting the completeness of the neuro-ophthalmologic assessment.

Small-vessel ischemic disease remains an important competing explanation, particularly given the chronic white matter changes reported on MRI. CTA did not demonstrate significant large-vessel occlusion, arterial dissection, or hemodynamically significant stenosis, and electrocardiography demonstrated sinus rhythm. However, prolonged cardiac rhythm monitoring, dedicated lower-extremity or pelvic venous imaging, and a complete hypercoagulability evaluation were not documented in the available records. These limitations prevent complete exclusion of alternative mechanisms and further support a cautious interpretation of the presumed stroke etiology.

The Risk of Paradoxical Embolism (RoPE) score and PFO-Associated Stroke Causal Likelihood (PASCAL) classification provide additional context for assessing the relationship between a PFO and cryptogenic stroke [2,13,14]. Based on the available records, the patient's age of 59 years, absence of hypertension or diabetes, nonsmoking status, absence of a documented previous stroke or transient ischemic attack (TIA), and noncortical pontine lesion yield a RoPE score of 6 [2,13]. Because the patient had an associated ASA, representing a high-risk PFO feature, the PASCAL classification places him in the "possible" PFO-associated stroke category [2,14]. This classification supports a potential but not definitive causal relationship.

Taken together, the patient's acute right INO, clinical diagnosis of a subacute pontine ischemic infarct, PFO with right-to-left shunting, associated ASA, and MTS provide a biologically plausible pathway for paradoxical embolism. However, MRI did not demonstrate an acute infarct, no active venous thrombus was documented, the etiologic evaluation had the limitations described above, and alternative ischemic mechanisms remain possible. Paradoxical embolism should therefore be regarded as a plausible but presumptive explanation rather than a proven mechanism.

## Conclusions

This case describes a clinically diagnosed subacute pontine ischemic infarct presenting predominantly with right INO, diplopia, and gait instability in a 59-year-old patient with a PFO, ASA, and MTS. Although MRI demonstrated an old pontine infarct without evidence of recent or acute infarction, neurology considered the acute clinical presentation consistent with a subacute pontine ischemic event.

The coexistence of a PFO with right-to-left shunting, high-risk PFO anatomy, and a potential venous thrombotic substrate provides a biologically plausible pathway for paradoxical embolism. However, no active venous thrombus was documented, and the causal relationship remains presumptive. This case emphasizes the importance of integrating the neurologic examination, neuroimaging, cardiac evaluation, and potential venous thrombotic sources when assessing otherwise unexplained brainstem ischemic syndromes.
